# Supplementary material for: A Non-Probiotic Fermented Soy Product Reduces Total and LDL Cholesterol: A Randomized Controlled Crossover Trial
Source: Nutrients. 2021 Feb 6;13(2):535. doi: 10.3390/nu13020535 (PMC7915103; doi:10.3390/nu13020535)
Supplement: Supplementary file 1 [file nutrients-13-00535-s001.pdf]

Table S1. Mean ratio between treatments of isoflavones in 24-h urine collected at the end of the intervention period.

	**Fermented Soy**	**Control**	**Mean ratio between treatments**	***P*-value**
Daidzein (μmol/d)	5.25 (2.29, 12.0)	0.48 (0.21, 1.11)	10.93 (5.07, 23.54)	<0.0001
Daidzein (μmol/g creatinine)	6.19 (2.69, 14.28)	0.56 0.24, 1.29)	11.17 (5.20, 24.00)	<0.0001
Genistein (μmol/d)	0.53 (0.24, 1.17)	0.24 (0.11, 0.52)	2.24 (1.14, 4.43)	0.0222
Genistein (μmol/g creatinine)	0.62 (0.29, 1.34)	0.27 (0.13, 0.59)	2.28 (1.20, 4.31)	0.0139
Glycitein (μmol/d)	0.62 (0.32, 1.23)	0.07 (0.04, 0.15)	8.48 (4.28, 16.80)	<0.0001
Glycitein (μmol/g creatinine)	0.73 (0.37, 1.47)	0.09 (0.04, 0.17)	8.64 (4.58, 16.30)	<0.0001

Mean ratio of Fermented Soy: Control. Values are marginal means (95% CI) adjusted for treatment, sequence, period, and enrollment period as fixed-effects terms and subjects as random-effects term. *P*-values of treatment main effect.

Table S2. Weighted means and SD of nutrient composition from two 24 h recalls obtained on fermented soy and two on control.

Nutrients	Fermented Soy (*n* = 24)[2]			Germinated Brown Rice (*n* = 24)[2]			Mean Differences between Fermented Soy and Germinated Brown Rice	P-value[3]
	Mean ± SD	Max	Min	Mean ± SD	Max	Min		
Energy (kcal)	1640 ± 480	2780	11.8	1790 ± 595	2650	410	−145	0.2499
Carbohydrate (g)	193 ± 68.2	323	74.4	229 ± 69.2	353	49.1	−36.1	0.0389
Protein (g)	65.2 ± 14.8	93.8	28.5	64.2 ± 26.4	144	20.5	0.99	0.8707
Animal protein (g)	33.1 ± 23.0	108.6	0.00	33.0 ± 24.7	108.6	1.13	−0.03	0.7900
Plant protein (g)	35.3 ± 11.9	55.8	18.5	37.2 ± 18.2	103.4	10.1	−1.84	0.6193
Fat (g)	70.3 ± 26.5	148	30.6	72.3 ± 33.1	139	14.4	−2.01	0.7678
Saturated fatty acids (g)	18.4 ± 8.27	40.3	6.76	21.8 ± 11.5	46.9	4.12	−3.50	0.3153
Monounsaturated fatty acids (g)	27.5 ± 10.9	56.7	9.60	27.1 ± 12.7	50.2	5.11	0.47	0.8703
Polyunsaturated fatty acids (PUFAs) (g)	19.0 ± 8.87	42.4	5.2	17.1 ± 10.0	52.9	2.43	1.93	0.2577
PUFAs 18:2 (linoleic acid) (g)	15.5 ± 8.03	36.1	2.42	14.7 ± 9.09	47.0	1.82	0.76	0.7281
PUFAs 18:3 (linolenic acid) (g)	1.83 ± 1.11	5.38	0.61	1.62 ± 1.0 1	5.06	0.12	0.21	0.3738
Cholesterol (mg)	195 ± 163	557	0.00	239 ± 177	650	6.49	−43.8	0.0320
Dietary fiber (g)	24.5 ± 9.47	44.8	11.9	24.3 ± 11.6	56.9	3.70	0.21	0.6139
Soluble dietary fiber (g)	5.76 ± 2.80	12.1	1.04	6.37 ± 3.22	17.4	0.83	-0.60	0.4316
Calcium (mg)	760 ± 457	1698	144	785 ± 423	1853	148	−25.5	0.6176
Iron (mg)	15.5 ± 5.80	32.7	9.27	13.7 ± 6.41	28.5	2.83	1.80	0.1294
Zinc (mg)	10.1 ± 2.91	17.5	5.92	9.18 ± 4.14	21.8	2.43	0.86	0.1775
Copper (mg)	1.70 ± 0.75	3.45	0.86	1.44 ± 0.72	3.72	0.16	0.27	0.0859
Magnesium (mg)	381 ± 132	601	185	335 ± 142	855	122	46.1	0.1117
Total soy isoflavones (mg)[4]	31.8 ± 14.5	44.8	0.14	13.4 ± 39.3	192	0.03	18.4	<.0001
Daidzein (mg)	12.5 ± 5.68	17.4	0.06	5.06 ± 15.1	73.4	0.09	7.43	<.0001
Genistein (mg)	16.19 ± 7.41	23.24	0.03	6.62 ± 19.95	97.13	0.001	9.57	<.0001
Glycitein (mg)	3.02 ± 1.39	4.27	0.00	1.26 ± 4.34	21.29	0.00	1.76	<.0001
Coumestrol (mg)	0.06 ± 0.09	0.35	0.00	0.08 ± 0.12	0.56	0.00	−0.01	0.6097
Biochania A (mg)	0.02 ± 0.06	0.21	0.00	0.34 ± 1.52	7.45	0.00	−0.32	0.2924
Formononetin (mg)	0.01 ± 0.02	0.12	0.00	0.00 ± 0.00	0.01	0.00	0.01	0.3100

[1]Dietary intake data were collected and analyzed using the Nutrition Data System for Research software, version 2018, developed by the Nutrition Coordinating Center, University of Minnesota, Minneapolis, MN.

[2]Calculated as [(weekday x 5) + (weekday x 2)]/7

[3]P-values of two-sample t-tests. Variables with skewed distributions were log transformed then back transformed. (SAS version 9.4, SAS Institute, Cary, NC)

[4]Sum of daidzein + glycitein + genistein + coumestrol + biochania A + formononetin
